# A protocol for a comparative evaluation of the fracture resistance of endodontically treated teeth reinforced with Cention N, resin-modified glass ionomer cement (RMGIC) and short fiber reinforced flowable composite as an intraorifice barrier

**DOI:** 10.12688/f1000research.133800.2

**Published:** 2024-07-26

**Authors:** Namrata Jidewar, Manoj Chandak

**Affiliations:** 1 Department of Conservative Dentistry and Endodontics, Sharad Pawar Dental College, Datta Meghe Institute of Higher Education and Research, Nagpur, Maharashtra, India

**Keywords:** dentistry, endodontics, Intraorifice barrier, post endodontic restoration, “Cention N”, “Resin modified glass ionomer cement”, “short fiber reinforced flowable composite”

## Abstract

**Background:**

Endodontic treatment is the most common method for resolving pulpal and periapical pathology. However, various studies have reported that almost 11%–13% of all teeth that undergo extraction after endodontic treatment show the presence of cracks, craze lines, and vertical root fractures. Teeth with inadequate post endodontic restoration are more prone to fracture and coronal leakage, resulting in the diffusion of oral fluids, bacteria, bacterial products, and possibly root canal treatment failure. Furthermore, studies have advocated the use of endodontically treated teeth with restorative materials that have a similar or higher elastic modulus than the tooth for providing stiffness against forces that cause root fracture. Intraorifice barriers made of restorative materials that can bond to radicular dentin could thus be used to reinforce the radicular dentin while also preventing coronal microleakage. Although the sealing ability of intraorifice barriers has been widely compared in the literature, there have been few studies on the strengthening effect of the materials used in the study as intraorifice barriers when placed into the root canal. As a result, the current *in vitro* study aims to assess the effect of various materials as intraorifice barriers (Cention N, Resin modified glass ionomer cement, and short fiber reinforced flowable composite) on the force required fracture teeth after root canal treatment.

**Methods:**

This *in vitro* study will be done on extracted human mandibular premolars with single root canal where after doing root canal treatment 2-3 mm obturating material would be replaced by intra orifice barriers (Cention N, resin modified glass ionomer cement [RMGIC], and short fiber reinforced flowable composite). The force required to fracture teeth will be calculated using universal testing machine.

## Introduction

Endodontic treatment is the primary approach for the resolution of pulpal and periapical pathology. However, various studies have reported that almost 11%–13% of all teeth that undergo extraction after endodontic treatment show the presence of cracks, craze lines, and vertical root fractures. ^1^ It is well-known that teeth with inadequate post endodontic restoration are more prone to fracture and coronal leakage, causing diffusion of oral fluids, bacteria, bacterial products, and possibly root canal treatment failure. ^2^ Irrigating solutions and intra canal medicaments used during chemico-mechanical preparation of root canal can alter collagen structure, which contributes to alteration of mechanical properties of dentin, resulting in fatigue crack propagation and hence increasing its susceptibility to vertical root fracture. ^2^ The concept of intraorifice barrier placement was first given by Roghanizad and Jones for prevention of microleakage. ^3^ Roghanizad and Jones, in order to reduce leakage, first proposed replacement of 3-mm of gutta-percha with restorative material at the root canal orifice. ^1^ Moreover, studies have advocated the use of restorative materials for endodontically treated teeth which have a similar or higher elastic modulus than the tooth can be proposed for providing stiffness against forces that generate root fracture. ^1^ Thus, intraorifice barriers with restorative materials that could bond to radicular dentin could be used for additionally reinforcing the radicular dentin as well as preventing coronal microleakage. Although the intra-orifice barriers was compared in terms of sealing ability in the literature widely, however there are limited studies in respect to strengthening effect of the materials which are being included in the study as intra orifice barriers when placed into the root canal. ^4^

As a result, the current study aims to assess the effect of various materials as intraorifice barriers (Cention N, Resin modified glass ionomer cement, and short fibre reinforced flowable composite) on the force required fracture teeth after root canal treatment.

## Objectives

1. To evaluate “fracture resistance” of endodontically treated teeth reinforced with Cention N as an intra orifice barrier.
2. To evaluate “fracture resistance” of endodontically treated teeth reinforced with RMGIC (resin modified glass ionomer cement) as an intra orifice barrier.
3. To evaluate “fracture resistance” of endodontically treated teeth reinforced with short fiber reinforced flowable composite as an intra orifice barrier.
4. To compare “fracture resistance” of endodontically treated teeth reinforced with Cention N, RMGIC, short fiber reinforced flowable composite as an intra orifice barrier.

### Protocol

*Study setting*

This *in vitro* study will be performed in the department of conservative dentistry and endodontics at Sharad Pawar Dental College in collaboration with central research laboratory (centre of translational sciences), Sawangi wardha.

*Sample selection*

This is an *in vitro* study and the sample required are human mandibular premolar extracted teeth. The patients whose treatment plan would include extraction of the teeth will be prior informed about the use of their extracted teeth for the study purpose and consent will be taken.

*Inclusion criteria*

• Teeth freshly extracted for orthodontic reasons with single canal and <10° curvature
• Noncarious human mandibular premolar teeth will be selected
• A vernier caliper will be used to measure the mesiodistal and buccolingual diameters of coronal aspect. A deviation up to ±10% from these mean values (mean = sum of all the values of mesio distal diameter of teeth/total number of extracted teeth to be used in the study) obtained will be considered as the sample

*Exclusion criteria*

• Teeth with cracks or crack lines from stereomicroscope (stereo zoom microscope ASI-ZOOM-I) evaluation (10×) will be discarded
• Teeth with more than one canal, existing caries, open apices, and curvature of roots more than 10° and with mesiodistal and buccolingual diameter of coronal plane exhibiting more than 10% difference from average will be excluded
• Radiographic assessment will be taken of the extracted teeth and it will be checked to ensure that the tooth has one canal. Teeth having internal and/or external resorption will be excluded

### Materials required

1. Resin modified glass ionomer cement (3M EspeVitremer) distributor – ayushi dentistry catalogue number – AD00366
2. Cention N (an alkasite group restorative material) (ivoclar viva dent) distributor – dentganga, catalogue number – DGIN21091-7
3. Short fiber reinforced flowable composite (Gc EverX Flow) distributor – get me dental, catalogue number – GCEVERXFO01
4. Diadent dia-proseal root canal sealer distributor – dentganga, catalogue number – DGIN220122-5947

### Distribution of samples

Group 1 - Cention N

Group 2 - Resin modified glass ionomer cement

Group 3 - short fiber reinforced flowable composite

### Sample preparation and method

For sample preparation, 33 intact mandibular premolar human teeth of similar dimensions will be selected for this study.

Specimens will be standardized to the length of 14 mm using a measuring scale. and decoronated by using diamond disc and water coolant. Patency and working length to be determined by #10K file. The canals will be biomechanically prepared with “rotary ProTaper Universal system (Dentsply)” till F3 using the crown-down technique (it is a technique of root canal preparation). ^5^

During instrumentation, canal irrigation will be done with 2 ml of 5% NaOCl and distilled water after each file change and lastly with a rinse of 5 ml of 17% EDTA. Obturation will be performed using the warm vertical compaction technique with corresponding gutta-percha points and diadent dia-proseal sealer.

The teeth will be assigned to four groups ( *n* = 11) by random sampling for the placement of intraorifice barriers. Except for the control group, all specimens will be prepared by removing coronal 3 mm of gutta-percha with use of gates glidden drill and the depth to be verified using periodontal probe.

Group 1: 4-Cention N (ivoclar) powder and liquid will be manipulated according to manufacturer’s instructions and placed in the cavity. It will be light-cured (woodpecker Light cure LED Mini-S) for 30 s.

Group 2: RMGIC (Vitremer, *3M ESPE, USA*), Shade A3. The primer will be applied with an applicator tip for 30 s to dentin and air dried. The primer will be then light cured for 20 s. After manipulating according to the manufacturer’s instructions, the material will be placed into the cavity, condensed and light-cured for 40 s.

Group 3: Short fiber reinforced flowable composite (Gc EverX Flow). The primer will be applied with a brush for 30 s to dentin and air dried. The primer will be then light cured for 20 s. the composite will be then placed into the cavity and light cured for 20 s.

After placing the intraorifice barrier materials, all specimens will undergo (With thermal and mechanical loading of 5000 cycles, 5 ± 2°C to 55 ± 2°C, dwell time 30 seconds, and 150,000 cycles at 60N) and stored at 37°C and at 100% humidity for 1 week in an incubator. The apical root end of each tooth will be mounted vertically along the long axis in self-curing acrylic resin such that 3 mm of each root will be exposed.

In order to acquire periodontal ligament simulation as in a tooth socket, light body elastomeric impression material will be used between the acrylic mold surface and tooth.

The specimens will be mounted on a Computerized Universal Testing Machine mechatronic UTE-20 (mechatronic in CRL DMIHER, wardha) and force will be applied along the long axis of roots on the canal with the velocity of 1 mm/min until fracture occurs. The force upon the sample breaking will be recorded in Newton.

### Sample size

The sample size was calculated using the following formula using mean difference

$$n1=n2=2\frac{{\left(Z_{\alpha }+Z_{\beta }\right)}^{2}\sigma^{2}}{{\left(\delta \right)}^{2}}$$

$$Z_{\alpha }=1.96$$

α=Type I error at5%

$$Z_{\beta }=1.28\;\left(1-\beta \right)=\text{Power at}\;90\%$$

σ=std. dev

Primary variable:- fravture resistance in newton

Fracture resistance in cention N group = 734.10 (As per Reference article)

Fracture resistance in RMGIC group = 491.60 (As per Reference article)

mean Difference = 242.5 (As per Reference article)

std. dev = 170.04 (As per Reference article)

As per reference articles.

$$\text{N1}=2^{*}\left[{\left(1.96+1.28\right)}^{2}{\left(170.04\right)}^{2}\right]/{\left(242.5\right)}^{2}=11$$

Total samples required = 11 per group.

### Statistical analysis

All the results will be calculated using SPSS version 27 software. Data for outcome variables will be tested for normality using kalmogorov-smirnov. The comparative analysis over the outcome fracture resistance between three groups (Cention-N, RMGIC, short fiber reinforced flowable composite) will be evaluated on the measurement of fracture resistance for finding significant difference on mean. ANOVA will be used to find the significant difference on mean between 3 groups. Tukey test will be used for comparative evaluation of measurement in between 2 groups pairwise. P-value ≤ 0.05 will be considered as significant at 5% level of significance and 95% confidence of interval.

### Dissemination

Advancements in dental materials will help broaden the perspective to have a holistic approach on using an intraorifce barrier thereby leading to increased strength of teeth which have undergone endodontic treatment. The outcome of this study will help clinicians to choose the best and most efficient filling materials that will increase the fracture resistance of endodontically treated teeth when used as an intraorifice barrier.

### Study status

Not started yet.

### Ethical considerations

Ethical approval was received from the ethical committee of Datta Meghe Institute of Higher Education and Research, Sawangi, Wardha (IEC reference number- DMIHER (DU)/IEC/2023/580) on 06/02/2023.

Written informed consent will be taken from patients who undergo extraction regarding the use of extracted teeth for the study purpose.

## Discussion

Root canal therapy has enabled dentistry to save teeth that would have been extracted without hesitation just a few decades ago. Endodontically treated teeth, on the other hand, are presumed to be more prone to fracture than vital teeth. After the completion of endodontic treatment, restoration and protection of the remaining tooth structure is of immence importancey. ^6^ Procedures performed after endodontic treatment has gotten more attention, as has their impact on the prognosis of non-vital teeth. But there is no significant difference in moisture content found between endodontically treated teeth and vital teeth. ^7^ The loss of tooth structure is most important in determining the prognosis of the endodontically treated teeth and reinforcing it with an adequate post endodontic restoration along with and intraorifice barrier of high strength and elastic modulus similar to that of dentin is of crucial importance. ^1^ Several studies have shown that force applied along the long axis of the tooth transmits the force uniformly and henceforth in the present study also the force be applied vertically along the long axis of the tooth. ^8^ The materials to be used in the study have properties like high strength and modulus of elasticity near to that of dentin and therefore they will be compared as intraorifice barrier materials in this study. RMGIC (resin modified GIC) was introduced in the late 1980s and contains some methacrylate components found in resin composites. Tselnik et al. reported superior performance as an acceptable coronal seal due to the better performance of RMGIC explained by water sorption by the material, which results in setting expansion and thus a better seal is achieved. It does not require dentin pretreatment and can adhere to it. Another useful property of “RMGIC” is fluoride release. Resin modified GIC has a high flexural strength and modulus of elasticity, and modulus of elasticity values similar to dentin, so the material can withstand a lot of stress before transmitting the load to the root… ^9^^–^^11^ Cention N has modulus of elasticity 13 Gpa. It also has patented isofiller which acts as shrinkage stress reliver thus, it helps to relives polymerization shrinkage. It also bonds to tooth structure micromechanically. Isofiller that leads to increased microhardness because filler particles are of nanoparticle size. It helps to withstand stresses and strains of the oral cavity. It can also be placed conservatively thus, reinforcing the remaining tooth structure. ^12^^,^^13^ In 2019, the latest type of flowable short fiber reinforced composite (everX Flow, GC, Tokyo, Japan) was launched globally. Several *invitro* studies have revealed that some SFRCs outperform conventional composites in terms of mechanical and physical performance. Short fibres incorporated in the matrix have significantly improved the material’s ability to resist crack propagation and also lowering the stress intensity at the tip of the crack and it’s propogation in an unstable manner and increase infracture toughness. Many of the properties of fiber-reinforced composites are dependent on microstructural parameters such as fiber diameter, fiber length, fiber orientation, fiber loading, and adhesion of fibers to the polymer matrix. ^14^^,^^15^

## Data Availability

No data is associated with this article.
