# Supplementary material for: A protocol for a comparative evaluation of the fracture resistance of endodontically treated teeth reinforced with Cention N, resin-modified glass ionomer cement (RMGIC) and short fiber reinforced flowable composite as an intraorifice barrier
Source: F1000Res. 2024 Jul 26;13:49. Originally published 2024 Jan 8. [Version 2] doi: 10.12688/f1000research.133800.2 (PMC11287110; doi:10.12688/f1000research.133800.2)
Supplement: Peer review history

# Reviewer report: Reviewer response for version 2

**DOI:** 10.5256/f1000research.167067.r307424

**Published:** 2024-07-29

**Authors:** Girija S. Sajjan

**Affiliations:** 1 Vishnu Dental College and Hospital, Andhra Pradesh, India

Okay. The design is approved.

Is the study design appropriate for the research question?

Partly

Is the rationale for, and objectives of, the study clearly described?

Yes

Are sufficient details of the methods provided to allow replication by others?

Partly

Are the datasets clearly presented in a useable and accessible format?

Not applicable

Reviewer Expertise:

Micro-endodontics, Guded endodontics, Regeneration, aesthetic dentistry, adhecive dentistry

I confirm that I have read this submission and believe that I have an appropriate level of expertise to confirm that it is of an acceptable scientific standard.

# Reviewer report: Reviewer response for version 1

**DOI:** 10.5256/f1000research.146813.r271206

**Published:** 2024-05-25

**Authors:** Girija S. Sajjan

**Affiliations:** 1 Vishnu Dental College and Hospital, Andhra Pradesh, India

1- Obturation will be performed using the single cone technique with corresponding gutta-percha points and diadent dia-proseal sealer.

 As most of the premolar pulp space will be not circular in cross-section, warm vertical compaction would be better.

 2-  All specimens will be prepared by removing coronal 3 mm of gutta-percha with a spoon excavator heated on a Bunsen burner.

 The removal can be done using TouchNheat equipment  with a tip or GG drill would be for better for gp removal rather than a spoon excavator

 3- All specimens will be stored at 37°C and at 100% humidity for 1 week in an incubator. 

 Thermal and mechanical loading of the specimen  will simulate the oral condition and are indicated.

Is the study design appropriate for the research question?

Partly

Is the rationale for, and objectives of, the study clearly described?

Yes

Are sufficient details of the methods provided to allow replication by others?

Partly

Are the datasets clearly presented in a useable and accessible format?

Not applicable

Reviewer Expertise:

Micro-endodontics, Guded endodontics, Regeneration, aesthetic dentistry, adhecive dentistry

I confirm that I have read this submission and believe that I have an appropriate level of expertise to confirm that it is of an acceptable scientific standard, however I have significant reservations, as outlined above.

# Response

**Published:** 2024-06-11

**Authors:** Namrata Jidewar

**Affiliations:** SHARAD PAWAR DENTAL COLLEGE, DATTA MEGHE INSTITUTE OF HIGHER EDUCATION, India

Respected ma'am

 Thank you so much for your valuable suggestions. I have incorporated them in my manuscript to the best of my knowledge and updated the same.

 Thank You,

 Regards,

 Dr. Namrata Jidewar

# Reviewer report: Reviewer response for version 1

**DOI:** 10.5256/f1000research.146813.r240458

**Published:** 2024-02-20

**Authors:** Pallav Mahesh Patni

**Affiliations:** 1 Department of Conservative Dentistry and Endodontics, Sri Aurobindo University, Indore, Madhya Pradesh, India

Abstract is well written and concise. 

 Introduction is clear and to the point.

 Materials and method are well elaborated and descriptive. 

 Results and Discussion are well explained.

 Overall, the manuscript contains several sections that could be clarified for better readability and coherence. Additionally, there are some minor errors in grammar, punctuation, and formatting throughout the text that should be addressed.

Is the study design appropriate for the research question?

Yes

Is the rationale for, and objectives of, the study clearly described?

Yes

Are sufficient details of the methods provided to allow replication by others?

Yes

Are the datasets clearly presented in a useable and accessible format?

Yes

Reviewer Expertise:

Endodontics, Dentistry, Conservative Dentistry.

I confirm that I have read this submission and believe that I have an appropriate level of expertise to confirm that it is of an acceptable scientific standard.

# Response

**Published:** 2024-06-11

**Authors:** Namrata Jidewar

**Affiliations:** SHARAD PAWAR DENTAL COLLEGE, DATTA MEGHE INSTITUTE OF HIGHER EDUCATION, India

Respected Sir,

 Thank you so much Sir.

 Regards,

 Dr. Namrata Jidewar
